# RASSF10 suppresses colorectal cancer growth by activating P53 signaling and sensitizes colorectal cancer cell to docetaxel

**DOI:** 10.18632/oncotarget.2866

**Published:** 2015-01-31

**Authors:** Jing Guo, Yage Yang, Yunsheng Yang, Enqiang Linghu, Qimin Zhan, Malcolm V. Brock, James G. Herman, Bingyong Zhang, Mingzhou Guo

**Affiliations:** ^1^ Department of Gastroenterology & Hepatology, Chinese PLA General Hospital, Beijing 100853, P.R.China; ^2^ Department of Gastroenterology and Hepatology, The First Affiliated Hospital of Zhengzhou University, Zhengzhou, Henan 450003, P.R.China; ^3^ State Key Laboratory of Molecular Oncology, Cancer Institute and Hospital, Chinese Academy of Medical Sciences & Peking Union Medical College, Beijing 100021, P.R.China; ^4^ Sidney Kimmel Comprehensive Cancer Center, Johns Hopkins University, Baltimore, Maryland 21231, U.S.A; ^5^ Department of Gastroenterology and Hepatology, Henan Provincial People's Hospital, Zhengzhou, Henan 450003, P.R.China

**Keywords:** RASSF10, DNA methylation, P53 signaling, colorectal cancer, epigenetics

## Abstract

RASSF10 has previously been reported to be frequently methylated in a number of malignancies. To understand the importance of RASSF10 inactivation in colorectal carcinogenesis, eight colorectal cancer cell lines, 89 cases of primary colorectal cancer and 5 cases of normal colorectal mucosa were examined. Methylation specific PCR, western blot, siRNA, gene expression array and xenograft mice were employed. The expression of RASSF10 was regulated by promoter regional methylation in colorectal cancer cells. RASSF10 was methylated in 60.7% (54/89) of primary colorectal cancers and was positively associated with tumor stage (*p* < 0.05) and metastasis (*p* < 0.05). Restoration of RASSF10 led to inhibition of colorectal cancer cell proliferation *in vitro* and *in vivo* and increased apoptosis. Gene expression arrays discovered RASSF10 inhibition of MDM2 expression as a mediator of these effects, which was confirmed with RT-PCR and western blot. RASSF10 was shown to activate P53 signaling in RKO and HCT116 cells after UV exposure, and sensitized these cells to docetaxel. In conclusion, our study demonstrates RASSF10 is frequently methylated in human colorectal cancer leading to loss of expression. RASSF10 normally suppresses human colorectal cancer growth by activating P53 signaling in colorectal cancer, and restored expression sensitizes colorectal cancer to docetaxel.

## INTRODUCTION

Colorectal cancer (CRC) is the third most common malignancies and the fourth most common cause of cancer related death in the world. About 1.2 million new cases were diagnosed and over 600 000 patients were died each year [1]. The pathogenesis of colorectal cancer remains unclear. Accumulation of gene mutation and epigenetic changes are regarded as important factors for the development of this malignancy [2–5]. Epigenetic changes can complement mutations to alter signaling pathways and are of interest-since they are potentially reversible with epigenetic therapy [6–8].

RASSF proteins were originally designated on the basis of sequence homology to domains that associated with Ras-like small GTP-binding proteins. These domains are known as Ras association (RA) domains and are distinct from Ras-binding domains (RBD) which bind an alternative set of Ras effectors. There are currently 10 members within the RASSF family (RASSF1–10) subdivided into two subgroups, the classical RASSF proteins (RASSF1–6) and the N-terminal RASSF proteins (RASSF7–10) based on the location of the RA domain [9, 10]. In addition to a RA domain, classical RASSF proteins also have a protein-protein interaction motif known as the SARAH (SARAH: SAlvador, RAssf, Hippo) domain that is responsible for scaffolding and regulatory interactions. The N-terminal RASSFs lack an identifiable SARAH domain, although the SMART database predicts that RASSF7, 8 and 10 contain extensive coiled-coil regions, which can be dimerized [11, 12]. RASSF10 gene is located on chromosome 11p15.2 and contains an over 2 kb CpG island in the promoter region [13]. RASSF10 is frequently methylated in different cancers [14–20]. Knockdown of RASSF10 increases mitosis in A549 lung cancer cells [11]. However, the epigenetic change and the function of RASSF10 in colorectal cancer remains unclear.

## RESULTS

### RASSF10 expression was regulated by promoter region hypermethylation in colorectal cancer cells

**Figure 1:**
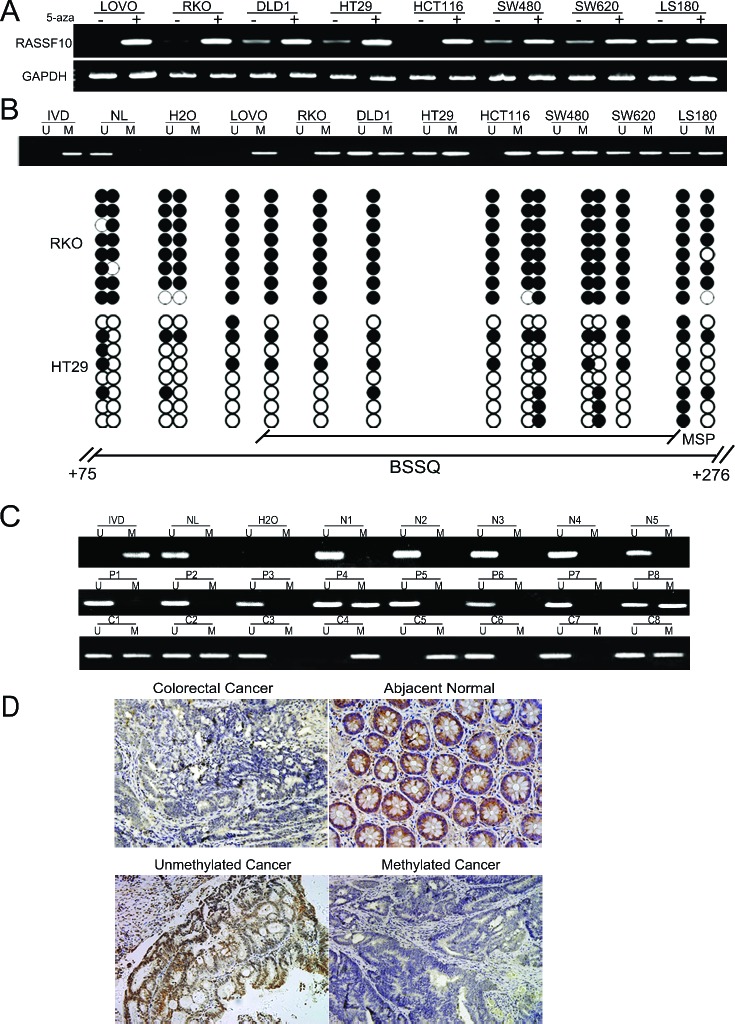
RASSF10 expression is regulated by promoter region hypermethylation in colorectal cancer **(A)** Expression of RASSF10 was detected by semi-quantitative RT-PCR. 5-aza: 5-aza-2′-deoxycytidine; GAPDH: The internal control of RT-PCR. DLD1, HT29, SW480, SW620, LOVO, RKO, HCT116 and LS180 are colorectal cancer cell lines. (–): absence 5-aza, (+): presence 5-aza. **(B)** Upper: RASSF10 MSP results in colorectal cancer cell lines. U: unmethylated alleles, M: methylated alleles; IVD: *In vitro* methylated DNA, serve as methylation control; NL: normal peripheral lymphocytes DNA, serve as unmethylation control; H2O: water. Down: Bisulfite sequencing (BSSQ) of RASSF10 promoter region (+75bp to +276bp) in RKO and HT29 cell lines. The region of CpG island studied by MSP is indicated by a horizontal line marked with MSP, spanning 114 bp. The region of CpG island studied by BSSQ is indicated by a horizontal line marked with BSSQ. filled circle: methylated CpG site; open circle: unmethylatedCpG site. **(C)** Upper: RASSF10 MSP results detected in normal human colorectal mucosa. N: normal colorectal mucosa. Middle: Representative MSP results of RASSF10 in human primary colorectal cancer adjacent tissue samples. P: adjacent tissue samples. Down: Representative MSP results of RASSF10 in human primary colorectal cancer. C: human primary colorectal cancer. **(D)** IHC results of RASSF10 expression in colorectal cancer and adjacent tissue samples (200×, upper), and the expression of RASSF10 in unmethylated and methylated colorectal cancer (200×, down).

To determine the level of expression of RASSF10, semi-quantitative RT-PCR was employed in colorectal cancer cells (LOVO, RKO, DLD1, HT29, HCT116, SW480, SW620, LS180). Expression of RASSF10 was found in cell line LS180, loss of RASSF10 expression was found in LOVO, RKO and HCT116 cells, and reduced expression was found in DLD1, HT29, SW480 and SW620 cells (Fig. 1A). As a test of whether RASSF10 expression was potentially affected by promoter region hypermethylation, these colorectal cancer cell lines were treated by 5-aza-2′-deoxycytidine (5-aza), a DNA methylation transferase inhibitor. Re-expression of RASSF10 was found in LOVO, RKO and HCT116 cells, and increased expression was found in LS180, DLD1, HT29, SW480 and SW620 cells (Fig. 1A), suggesting that DNA methylation may have led to transcriptional repression. Using methylation specific PCR (MSP) to examine the promoter region of RASSF10, complete methylation was found in LOVO, RKO and HCT116 cells, and partial methylation was found in LS180, DLD1, HT29, SW480 and SW620 cells (Fig. 1B). Thus, promoter region methylation correlated with loss/reduced RASSF10 expression. To further validate the efficiency of MSP primers and the methylation density in the promoter region, sodium bisulfite sequencing was performed in RKO and HT29 cell lines. The results demonstrate that MSP result is correlated with bisulfite sequencing results very well (Fig. 1B). Genomic sequencing was next performed to exclude the possibility of RASSF10 mutation causing loss or reduction of expression. One mutation was found in HCT116 cells at 591 locus (G to A) converts Arg to His. 2 different mutations were found in 2 of 89 cases primary colorectal cancer. One is at 806 +locus (G to C) converts Glu to Gln. Another is at 1550 locus (A to C) converts Met to Leu. However, none of these mutations explain the loss of RASSF10 expression and mutation of RASSF10 appears to be a rare event in human colorectal cancer. The results suggest that RASSF10 expression is mainly altered by promoter region methylation in colorectal cancer.

### RASSF10 was frequently methylated in human primary colorectal cancer

To determine whether methylation of RASSF10 occurred in primary colorectal cancer, 89 cases of colorectal cancer samples with paired normal tissue and 5 cases of normal colorectal mucosa were examined by MSP. 60.7% (54/89) of colorectal cancer and 26.9% (24/89) of adjacent tissue samples were methylated, while no methylation was found in normal colorectal mucosa (Fig. 1C). The methylation of RASSF10 was associated with lymph node metastases (*P* < 0.05) and late tumor stage (*P* < 0.05), but no association was found with age, gender, anatomic location and differentiation (Table 1). RASSF10 expression was next evaluated using immunohistochemistry in 27 available colorectal cancer and matched adjacent tissue samples. Without reduction of RASSF10 expression and unmethylation were found in 7 cases of cancer tissue samples. Reduced expression was found in 20 cases of colorectal cancer and 3 cases of adjacent tissue samples. The expression of RASSF10 was reduced significantly in colorectal cancer compared with adjacent tissue samples (*P* < 0.05) (Fig. 1D). In the 20 cases of cancer samples, which RASSF10 expression was reduced, 13 cases were methylated. Reduced expression was associated with RASSF10 promoter region hypermethylation significantly (*p* < 0.05). (Fig. 1D).

**Table 1:** Clinicopathological features and RASSF10 methylation in 89 patients with colorectal cancer

Clinical Factor	No.	RASSF10 methylation status		
		methylated *n* = 54(60.7%)	unmethylated *n* = 35(39.3%)	*P* value (χ^2^ test)
**Age**				
<60	50	32	18	0.611
≥60	39	22	17	
**Gender**				
M	60	36	24	0.965
F	29	18	11	
**Location**				
Left	33	22	11	0.730
Transverse	6	3	3	
Right	19	10	9	
Rectum	42	25	17	
**Metastases**				
Negative	45	21	24	0.012^*^
Positive	44	33	11	
**TNM stage**				
I–II	32	10	22	0.001^*^
III–IV	57	44	13	
**Differentiation**				
Poor	27	18	9	0.349
Moderate	60	34	26	
Well	2	2	0	

* *p* values were obtained from the chi-square test; significant difference, *P* < 0.05.

### Restoration of RASSF10 expression inhibits cell proliferation, induces apoptosis and G2/M phase arrest in CRC cells

**Figure 2:**
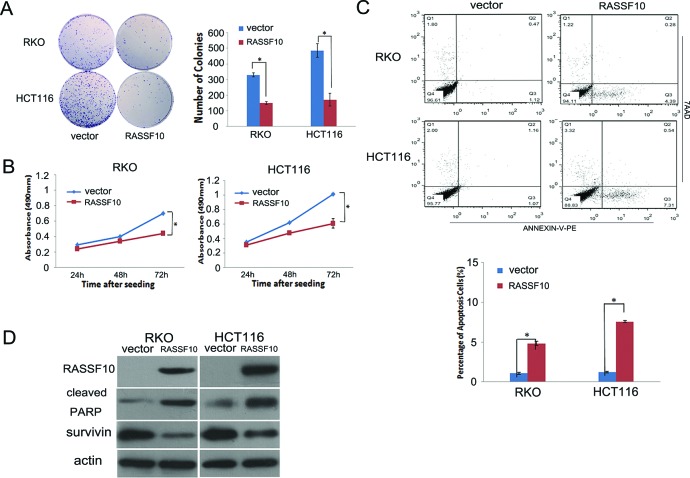
RASSF10 expression alters colorectal cancer cell proliferation and apoptosis **(A)** Representative results of colony formation. Blue bar: Represent RASSF10 unexpressed RKO and HCT116 cells; Red bar: Represent RASSF10 expressed RKO and HCT116 cells. The experiment was repeated for three times. *: *p* < 0.05. **(B)** Growth curves of RASSF10 expressed and unexpressed RKO and HCT116 cells analyzed by MTT assay. Points: three independent experiments. *: *p* < 0.05. **(C)** Percentage of apoptosis cells in RASSF10 unexpressed and expressed RKO and HCT116 cells. *: *p* < 0.05. **(D)** The expression of RASSF10, cleaved PARP and survivin detected by western blot in RASSF10 unexpressed and expressed RKO and HCT116 cells. Actin: internal control.

The effect of RASSF10 on cell proliferation was analyzed by colony formation, MTT and flow cytometry in RKO and HCT116 cells. There was a 55–65% reduction in colony formation, with the number of colonies being 329 ± 13 *vs* 149 ± 8 (*P* < 0.05) and 485 ± 44 *vs* 171 ± 41 (*P* < 0.05) before and after restoration of RASSF10 expression in RKO and HCT116 cells respectively (Fig. 2A). Cell viability was determined using MTT, with a 35% reduction, with OD values of 0.703 ± 0.047 *vs* 0.462 ± 0.039 (*P* < 0.05) in RKO cells and 1.031 ± 0.081 *vs* 0.680 ± 0.061 (*P* < 0.05) in HCT116 cells before and after restoration of RASSF10 expression (Fig. 2B). These results suggest that RASSF10 inhibits cell proliferation in colorectal cancer cells.

Next, we measured apoptotic cells after restoration of RASSF10 expression in RKO 4.84 ± 0.26% *vs* baseline of 1.07 ± 0.13% (*P* < 0.05), and HCT116 cells 7.55 ± 0.13% *vs* baseline of 1.22 ± 0.12% (*P* < 0.05) (Fig. 2C). To confirm the effect of RASSF10 on apoptosis in colorectal cancer cells, cleaved PARP and survivin, a representative apoptotic marker and anti-apoptotic marker, were examined by western blot before and after restoration of RASSF10. Expression of cleaved PARP was increased and expression of survivin was reduced after re-expression of RASSF10 in RKO and HCT116 cells (Fig. 2D), confirming the annexin staining of increased apoptosis in CRC cells.

**Figure 3:**
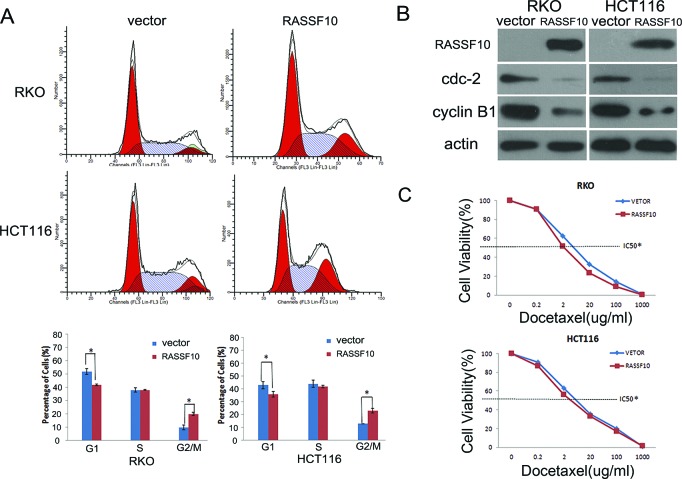
RASSF10 expression alters cell cycle regulations and sensitivity of colorectal cancer cells to docetaxel **(A)** Cell phase distribution in RASSF10 unexpressed and expressed RKO and HCT116 cells analysed by flow cytometry. *: *p* < 0.05. **(B)** The expression of RASSF10, CDC2 and cyclin B1 detected by western blot in RASSF10 unexpressed and expressed RKO and HCT116 cells. Actin: internal control. **(C)** The cell viability in RASSF10 unexpressed and re-expressed RKO and HCT116 cells after docetaxel treatment. IC50: the half maximal inhibitory concentration Points: three independent experiments. *: *P* < 0.05.

To further examine growth inhibition, we determined cell cycle distribution before and after restoration of RASSF10 expression in RKO cells with the following findings: 51.8 ± 2.3% *vs* 42.4 ± 0.7% (*P* < 0.05) in G0/1 phase, 37.9 ± 1.6% *vs* 38.1 ± 0.5% in S phase and 10.4 ± 1.8% *vs* 19.5 ± 1.2% (*P* < 0.05) in G2/M phase. The cell phase distribution before and after restoration of RASSF10 expression in HCT116 cells was as follows: 43.0 ± 5.7% *vs* 35.7 ± 4.4% (*P* < 0.05) in G0/1 phase, 44.2 ± 5.7% *vs* 41.6 ± 1.8% in S phase and 12.8 ± 0.1% *vs* 22.7 ± 2.7% (*P* < 0.05) in G2/M phase. This suggested that G0/1 phase was reduced and G2/M phase was increased in RKO and HCT116 cells (Fig. 3A), suggesting G2/M checkpoint inhibition. To further validate the effect of RASSF10 on G2/M check point, the expression of cdc-2 and cyclin B1, important G2/M check point regulators, was determined using western blot. The expression of cdc-2 and cyclin B1 was reduced after re-expression of RASSF10 in RKO and HCT116 cells (Fig. 3B), providing a mechanism by which RASSF10 induces G2/M arrest in colorectal cancer.

### Restoration of RASSF10 sensitizes RKO and HCT116 cells to docetaxel

Docetaxel is a microtubule inhibitor and is mainly targeting mitotic phase. As knockdown of RASSF10 increases mitosis in A549 lung cancer cells [11] and we found restoration of RASSF10 induces G2/M arrest in colorectal cancer. We explored the sensitivity of RKO and HCT116 cells to docetaxel before and after re-expression of RASSF10. The IC50 of docetaxel was 4.508 ± 0.175 *vs* 2.688 ± 0.103 μg/ml (*P* < 0.05) in RKO cells and 5.366 ± 0.043 *vs* 3.800 ± 0.201 μg/ml (*P* < 0.05) in HCT116 cells before and after restoration of RASSF10 expression (Fig. 3C). The results suggest that RASSF10 functions as a mitotic inhibitor in human colorectal cancer and sensitizes cancer cells to docetaxel.

### Isolation and identification of cancer related genes regulated by RASSF10 in colorectal cancer

**Figure 4:**
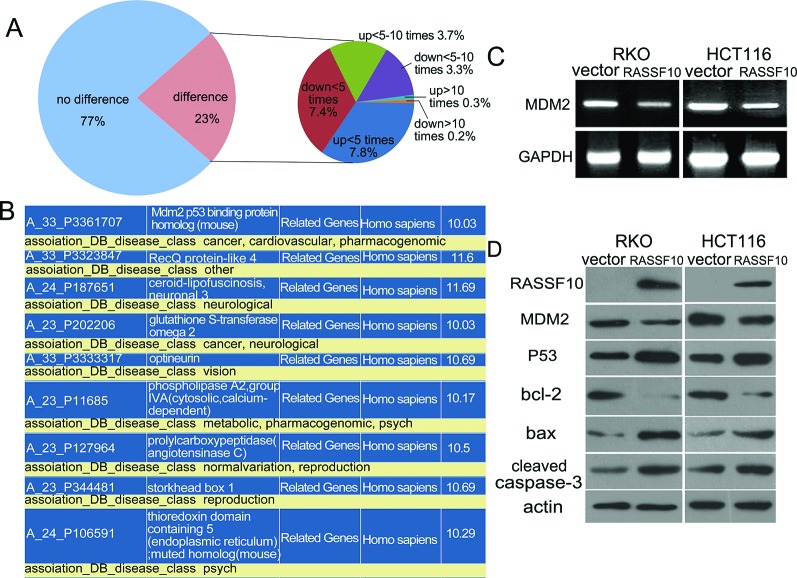
RASSF10 involved pathways in colorectal cancer **(A)** Differentially expressed genes in RASSF10 re-expressed and unexpressed RKO cells were analyzed by gene expression array. The percentage of up regulated or down regulated genes were shown in the pie chart. Angle of the sector: the percentage of each category. Totally, 81 genes were changed over 10 times after re-expression of RASSF10 in RKO cells. **(B)** Among the 81 genes, MDM2 and GSTO2 are cancer-related genes according to Disease-related gene analysis (http://david.niaid.nih.gov). **(C)** The expression of MDM2 was detected by semi-quantitative RT-PCR in RASSF10 unexpressed and expressed RKO and HCT116 cells. **(D)** The expression of MDM2, P53, bax, bcl-2 and cleaved caspase-3 was detected by western blot in RASSF10 unexpressed and expressed RKO and HCT116 cells. Actin: internal control.

To understand the mechanism by which RASSF10 functions to alter proliferation and apoptosis in colorectal cancer, gene expression microarray was utilized. The expression of 3575 genes were increased or reduced by more than two fold in RASSF10 re-expressed RKO cells. Among these genes, 46 were up regulated more than 10 fold and 35 were down regulated more than 10 fold (Fig. 4A). Further analysis found among these genes, only MDM2 and GSTO2 belong to cancer related genes according to Diseases Association Analysis (http://david.niaid.nih.gov) (Fig. 4B). Of greatest interest was MDM2, which is known to play an important role in the tumorigenesis as a key component of P53 signaling which directly promotes P53 degradation by catalyzing the ubiquitination of P53. Down regulation of MDM2 following restoration of RASSF10 expression was confirmed using RT-PCR (Fig. 4C) and further confirmed using western blot (Fig. 4D).

### RASSF10 activates P53 signaling in colorectal cancer

To determine the role of RASSF10 in P53 signaling, the expression of P53, bcl-2, bax and cleaved caspase-3 were examined using western blot in RASSF10 expressed and baseline RKO and HCT116 cells. The expression of P53, bax and cleaved caspase-3 all increased and bcl-2 was reduced following restoration of RASSF10 expression in RKO and HCT116 cells (Fig. 4D).

**Figure 5:**
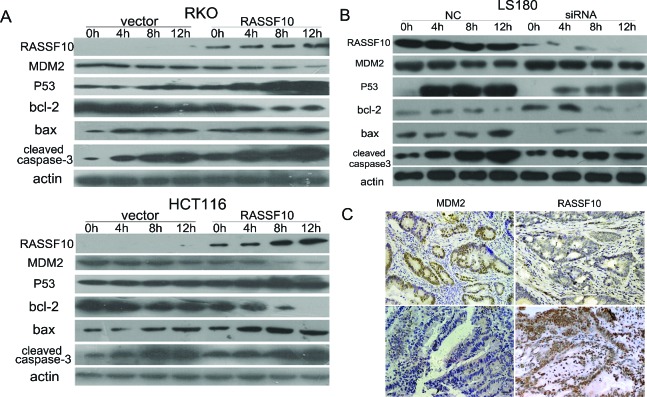
RASSF10 effects on P53 signaling **(A)** The expression of MDM2, P53, bax, bcl-2 and cleaved caspase-3 was detected by western blot in RASSF10 unexpressed and expressed RKO and HCT116 cells after UV exposure for 0 h, 4 h, 8 h and 12 h. Actin: internal control. **(B)** The expression of MDM2, P53, bax, bcl-2 and cleaved caspase-3 was detected by western blot in LS180 cells before and after RASSF10 knocking down collected at 0 h, 4 h, 8 h and 12 h after UV exposure. Actin: internal control. **(C)** Representative images of MDM2 and RASSF10 expression in human primary colorectal cancer tissues by IHC. (images, 200×).

To further study the function of RASSF10 in P53 signaling, DNA damage was induced by exposing RKO and HCT116 cells to UV. The expression of P53, bax and cleaved caspase-3 was increased, and MDM2 and bcl-2 were reduced only after re-expression of RASSF10 in UV exposed RKO and HCT116 (Fig. 5A). This further indicates that P53 signaling was activated by RASSF10 in colorectal cancer.

To confirm this effect of RASSF10 in P53 signaling, siRNA knockdown technique was employed in LS180 cells which basally express the highest level of RASSF10 among colorectal cancer cell lines. As shown in Fig. 5B, UV exposure increases the expression of P53, bax and cleaved caspase-3 in the parental cells which is diminished with knockdown of RASSF10. These results further suggest that RASSF10 is a modulator of P53 signaling pathway in human colorectal cancer.

### The expression of MDM2 was associated with RASSF10 expression in reversely in human primary colorectal cancer

The expression of MDM2 and RASSF10 was evaluated by immunohistochemistry in 27 available cases of human primary colorectal cancer. In 7 cases of human primary colorectal cancer with high level expression of RASSF10, the expression of MDM2 was diminished. In 20 cases with reduced RASSF10 expression, 17 cases had MDM2 high level expression while 3 cases had low level expression. The expression of MDM2 is inversely correlated with RASSF10 in primary colorectal cancer (Fig. 5C, *P* < 0.05).

### RASSF10 suppresses HCT116 cell xenograft growth in mice

**Figure 6:**
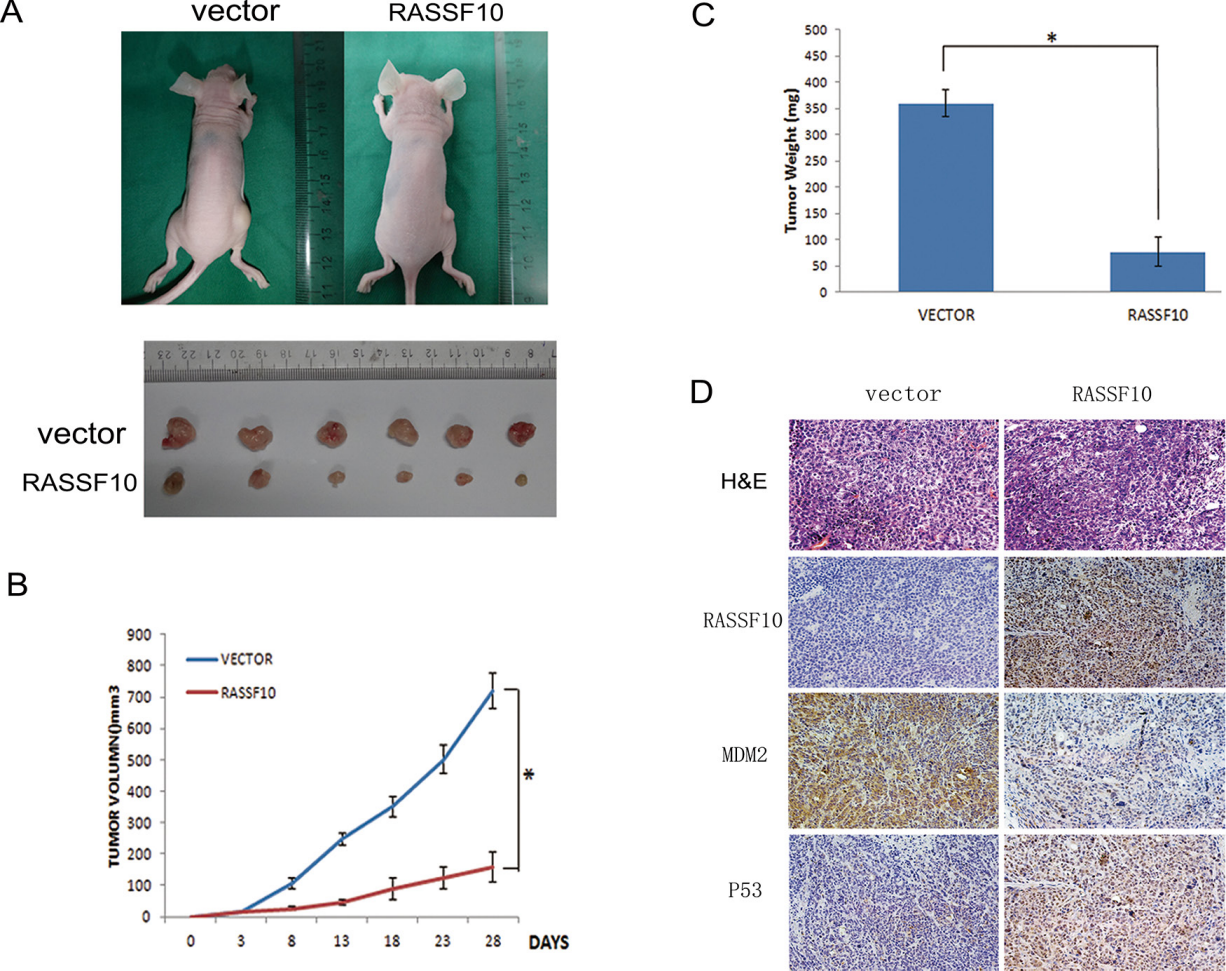
RASSF10 expression inhibits growth of HCT116 cell xenografts **(A)** Representative tumors of RASSF10 unexpressed and expressed HCT116 cell xenograft. **(B)** Tumor growth curves after injection of RASSF10 unexpressed and expressed HCT116 cells. Points: mean of 6 mice. *: *p* < 0.05. **(C)** Tumor weight in nude mice at the 28th day after inoculation of RASSF10 unexpressed and expressed HCT116 cells. Bars: mean of 6 mice. *: *p* < 0.05. **(D)** Images of H&E staining showing necrosis degree of tumor from RASSF10 expressed and unexpressed xenograft tumor. IHC staining to reveal the expression of RASSF10 in RASSF10 expressed HCT116 cell xenografts(right) and the empty vector transfected HCT116 cell xenografts (left). The expression of MDM2 and P53 was examined by IHC in xenografts. (images, 200×).

To further study the function of RASSF10 in colorectal cancer, RASSF10 expressed and unexpressed HCT116 cell xenograft mice models were employed (Fig. 6A). Tumor growth was marked diminished with RASSF10 expression, with the mean tumor volumes measured at 28 days being 721.25 ± 55.7 mm^3^ *vs* 158.17 ± 49 mm^3^ (*P* < 0.05, Fig. 6B), and the weight was 360 ± 25 mg *vs* 76.67 ± 27.5 mg (*P* < 0.05) in RASSF10 unexpressed and expressed HCT116 cell xenografts respectively (Fig. 6C). The expression of RASSF10, MDM2 and P53 in xenografts was examined by IHC. The expression of MDM2 was reduced and the expression of P53 increased in RASSF10 re-expressed HCT116 cell xenografts compared to parental controls not expressing RASSF10 (Fig. 6D).

## DISCUSSION

RASSF10 has been reported to be frequently methylated in different malignancies and has been regarded as tumor suppressor [14–20]. In this study, RASSF10 was found to be frequently methylated in human colorectal cancer with resulting loss of expression. The presence of methylation of RASSF10 was associated with adverse features including lymph node metastases (*P* < 0.05) and increased tumor stage (*P* < 0.05). Since we have demonstrated tumor suppressor functions for RASSF10, reversal of RASSF10 repression may potentially be an approach for colorectal cancer therapy. RASSF10 methylation may also serve as a marker of poor prognosis of colorectal cancer.

Our study provides a molecular mechanism by which RASSF10 acts as a tumor suppressor gene. Through the use of gene expression arrays, we identified genes regulated by RASSF10 directly or indirectly. This demonstrated down regulation of MDM2 by RASSF10 as a mechanism to activate p53. MDM2 is a key component of P53 signaling and directly promotes P53 degradation by catalyzing the ubiquitination of P53. MDM2 has been reported to play an important role in tumorigenesis and interacts with several members of the RASSF family directly or indirectly. For example, RASSF1A, RASSF3, RASSF5A and RASSF6 have been shown to interact with MDM2 and facilitate its degradation, resulting in the stabilization of P53 [21–24]. RASSF3 interacts with MDM2 via SARAH domain [22], while RASSF1A promotes MDM2 self-ubiquetination by disrupting the MDM2-DAXX-HAUSP complex, resulting in the stabilization and activation of p53 [22]. Similarities of RASSF10 to other RASSF proteins suggest that it could also activate P53 signaling by promoting MDM2 degradation. P53 plays a pivotal role in regulation of cellular processes including cell cycle arrest and apoptosis. Activating p53 signaling may either induce cell cycle arrest or inhibit cell growth and promote cell apoptosis [25–29]. Indeed, our study indicates that the expression of MDM2 was suppressed by restoring expression of RASSF10, and P53 signaling was activated in colorectal cancer cells. These results suggest that RASSF10 suppresses colorectal cancer cell growth by activating P53 signaling.

Previous studies in A549 lung cancer cells reported that knockdown of RASSF10 increases mitosis [11]. In this study we demonstrate that restoring RASSF10 expression induced both G2/M arrest and apoptosis in colorectal cancer cells. Since docetaxel is a microtubule inhibitor which is most effective in mitotic phase, we found that sensitivity of colorectal cancer cells to docetaxel was indeed increased in RKO and HCT116 when RASSF10 expression was restored.

In conclusion, RASSF10 was frequently methylated in human colorectal cancer and methylation of RASSF10 was associated with late tumor stage and metastases. The expression of RASSF10 was regulated by promoter region hypermethylation. RASSF10 suppressed human colorectal cancer by activating P53 signaling both *in vitro* and *in vivo*. RASSF10 is a mitosis inhibitor whcih sensitized colorectal cancer cells to docetaxel.

## MATERIALS AND METHODS

### Human tissue samples and cell lines

Eight human CRC cell lines (LOVO, DLD1, RKO, HT29, HCT116, SW480, SW620 and LS180) were included in this study. All the CRC cell lines were established from primary colorectal cancer previously and maintained in 90% RPMI 1640 (Invitrogen, CA, USA), supplemented with 10% fetal bovine serum and antibiotics.

A total of 89 cases of primary colorectal cancer were surgically resected and five cases of normal colorectal mucosa were collected from noncancerous patients by biopsy under endoscopy. All tissues were collected from Chinese PLA General Hospital according to the approved guidelines of the Chinese PLA General Hospital's institutional review board and kept at −80°C.

### 5-aza-2′-deoxycytidine (5-aza) and UV treatment

CRC cell lines were split to a low density (30% confluence) 12 h before treatment. Cells were treated with 5-aza (Sigma, St. Louis, MO, USA) at a concentration of 2 μM in the growth medium, which was exchanged every 24 h for total 96 h treatment. At the end of the treatment course, RNA was isolated as described below. Cells were cultured in 10 cm plates. Medium was removed and cells were exposed to 50 J/m^2^ of UVC (UV Cross-linker, China).

### RNA isolation and semi-quantitative RT-PCR

Total RNA was isolated by Trizol reagent (Invitrogen, Carlsbad, USA). RNA quality and quantity were evaluated by Agarose gel(1%) electrophoresis and spectrophotometric analysis. Semi-quantitative reverse transcription-PCR (RT-PCR) was performed as described previously [30]. RT-PCR primers are as follow: 5′-GTCGTCCTGTTCGTCCACTT-3′ (F), 5′-TGTCCTGCACGTAGTTGACC-3′ (R).

### DNA extraction, methylation-specific PCR (MSP) and bisulfite sequencing (BSSQ)

Genomic DNA of CRC cells and tissue samples were extracted by proteinase K method. MSP and BSSQ were performed as described previously [31, 32]. Primers for MSP and BSSQ were designed around transcription start site. The sequences of MSP primers as follow: 5′-GTGTTATGGATTTTTTGGAAAAGAAGATATT-3′ [33], 5′-TCCTCCAAAAACACTCACACAACATCA-3′ (UR), 5′-GTTATGGATTTTTCGGAAAAGAAGATATC-3′ (MF) and 5′-CTCCAAAAACACTCGCACAACGT CG-3′ (MR). Primers sequences of BSSQ are as follow: 5′-GAGTTATTGGGTTGTTTTTGTTG-3′ (F) and 5′-CRACAACCRTCCTCCAAAAAC-3′ (R).

### Immunohistochemistry (IHC)

Immunohistochemical staining was performed on 4 μm thick serial sections derived from formaldehyde-fixed paraffin blocks. Rabbit polyclonal antibody against RASSF10 (Abgent, San Diego, U.S.), MDM2 ( Abgent, San Diego, U.S.) and rabbit monoclonal antibody against P53 (Bioworld Tech, MN, USA) were employed. IHC was performed and evaluated as described previously [32]. The staining intensity and extent of the staining area were graded according to the German semi-quantitative scoring system as described before [34, 35]. Staining intensity of the nucleus, cytoplasm, and/or membrane was characterized as follows: no staining = 0; weak staining = 1; moderate staining = 2; strong staining = 3; the extent of staining was defined as: 0% = 0, 1–24% = 1, 25–49% = 2, 50–74% = 3, 75–100% = 4. The final immune-reactive score (0 to 12) was determined by multiplying intensity score with the extent of staining score.

### Plasmid construction and transfection

The RASSF10CDs region was amplified by RT-PCR. The primers are as follows: 5′-GATCGAATTCGCCA CCATGGATCCTTCGGAAAAGAAGATATC-3′ (F) and 5′-GATCTC TAGACTACACAAGGGATTCG CACATG-3′ (R). The products were subcloned into the lentiviral vector pLVX-IRES-ZsGreen1 (Clontech Laboratories, Inc, Takara U.S.). Then, the lentiviral RASSF10 plasmid was co-transfected into HEK 293T cells along with pVSVG and pΔ8.91 using PEI (cat: 23966 polysciences USA). Cells were infected with recombinant lentivirus-transducing units.

### SiRNA knockdown assay

SiRNA knockdown assay was performed according to the manufacturer's instructions. The sequences of siRNA targeting RASSF10 and RNAi Negative Control Duplex are as follow: siRNA duplex (sense: 5′-GCGAAGAGCAAGAGAAUGUTT-3′; antisense: 5′-ACAUUCUCUUGCUCUUCGCTT-3′); RNAi negative control duplex (sense: 5′-UUCUCCGAACGUGU CACGUTT-3′; antisense: 5′-ACGUGACACGUUC GGAGAATT-3′) (Gene Pharma Co, Shanghai, China).

### Colony formation assay

Cells were plated into 3.5 cm dishes and cultured for 2 weeks. Then cells were fixed with 75% ethanol for 30 minutes, stained with 0.2% crystal violet (Beyotime, Nanjing, China) for 20 minutes and counted.

### Cell viability assay

Cells were plated in 96-well plate at a density of 3 × 10^3^ cells/well, and cell viability was measured at 24, 48 and 72 h using the MTT assay kit (Promega, Madison, Wisconsin, USA) according to the company's instruction. Absorbance was measured on a microplate reader (Thermo Multiskan MK3 USA) at a wavelength of 490 nm.

For chemo-sensitivity assay, cells were seeded in 96-well plates and treated with docetaxel at the dose of 0, 0.2, 2, 10, 100 and 1000 μg/ml. Absorbance was measured as above. The percentage of viable cells (%) = (A490(treated) - A490(blank))/(A490(control) - A490(blank)) × 100%. IC50 was defined as the concentration, which was required for 50% inhibition of cell growth.

### Flow cytometry analysis of cell cycle and apoptosis

Cell cycle progression was examined as described in the previous article [31]. For apoptosis analysis, Annexin V-PE/7-AAD Apoptosis Detection Kit (KeyGen Biotechnology, China) was conducted according to manufacturer's instructions. Each sample was analyzed by flow cytometry with a FACScan Flow Cytometer (Becton-Dickinson Biosciences, Mansfield, MA).

### Microarray analysis

RNA from RASSF10 expressed and unexpressed RKO cells were purified using the RNeasy Mini Kit (QIAGEN China (Shanghai) Co., Ltd.) and then analyzed by gene expression array (Agilent Human (4 × 44k)).

### Western blot

Western blot was performed as described previously [30]. Antibodies were diluted according to manufacturer's instruction. Primary antibodies are as follow: RASSF10 (Abgent, San Diego, U.S.), MDM2 (Abgent, San Diego, U.S.), P53 (Bioworld Tech, MN, USA), cleaved PARP (Cell Signalinging Technology, USA), cleaved caspase-3 (Cell Signalinging Technology, USA), bax(Beyotime Biotech, China), bcl-2 (Cell Signalinging Technology, USA), cyclinB1 (Bioworld Tech, MN, USA), cdc-2 (Bioworld Tech, MN, USA), survivin (Bioworld Tech, MN, USA) and β-actin (Beyotime Biotech, China).

### The effect of RASSF10 on HCT116 cell xenograft

RASSF10 expressed and unexpressed HCT116 cells (3 × 10^6^ cells in 0.1 ml phosphate-buffered saline) was subcutaneously injected into the dorsal flank of 5-week-old female BABL/c nude mice (*n* = 6 each group). The tumor size was measured 3 days after inoculation for 4 weeks by every 5 days. The tumor volume was determined with the following formula: tumor volume (mm^3^) = (length (mm)) × (width (mm))^2^ / 2. All procedures were approved by the Animal Ethics Committee of the Chinese PLA General Hospital.

### Statistical analysis

SPSS 17.0 software was employed. Quantitative data were presented as the mean ± SEM. A *p* value of less than 0.05 was considered statistical significance.
